# The Benefit of Neuromuscular Blockade in Patients with Postanoxic Myoclonus Otherwise Obscuring Continuous Electroencephalography (CEEG)

**DOI:** 10.1155/2017/2504058

**Published:** 2017-02-06

**Authors:** Christopher R. Newey, Alejandro Hornik, Meziane Guerch, Anantha Veripuram, Sushma Yerram, Agnieszka Ardelt

**Affiliations:** ^1^Department of Neurology, University of Missouri, 5 Hospital Drive, CE 540, Columbia, MO 65211, USA; ^2^Southern Illinois Healthcare, 405 W. Jackson Street, Carbondale, IL 62902, USA; ^3^Novant Health Forsyth Medical Center, 3333 Silas Creek Parkway, Winston-Salem, NC 27103, USA; ^4^Department of Neurology, Texas Tech University Health Sciences Center-El Paso, 4615 Alameda Avenue, El Paso, TX 79905, USA; ^5^Department of Neurology, The University of Chicago Medicine, 5841 S. Maryland Drive, MC2030, Chicago, IL 60637, USA

## Abstract

*Introduction*. Myoclonus status epilepticus is independently associated with poor outcome in coma patients after cardiac arrest. Determining if myoclonus is of cortical origin on continuous electroencephalography (CEEG) can be difficult secondary to the muscle artifact obscuring the underlying CEEG. The use of a neuromuscular blocker can be useful in these cases.* Methods*. Retrospective review of CEEG in patients with postanoxic myoclonus who received cisatracurium while being monitored.* Results*. Twelve patients (mean age: 53.3 years; 58.3% male) met inclusion criteria of clinical postanoxic myoclonus. The initial CEEG patterns immediately prior to neuromuscular blockade showed myoclonic artifact with continuous slowing (50%), burst suppression with myoclonic artifact (41.7%), and continuous myogenic artifact obscuring CEEG (8.3%). After intravenous administration of cisatracurium (0.1 mg–2 mg), reduction in artifact improved quality of CEEG recordings in 9/12 (75%), revealing previously unrecognized patterns: continuous EEG seizures (33.3%), lateralizing slowing (16.7%), burst suppression (16.7%), generalized periodic discharges (8.3%), and, in the patient who had an initially uninterpretable CEEG from myogenic artifact, continuous slowing.* Conclusion*. Short-acting neuromuscular blockade is useful in determining background cerebral activity on CEEG otherwise partially or completely obscured by muscle artifact in patients with postanoxic myoclonus. Fully understanding background cerebral activity is important in prognostication and treatment, particularly when there are underlying EEG seizures.

## 1. Introduction

Out-of-hospital cardiac arrest remains a major cause of morbidity and mortality with only approximately 50% of cardiopulmonary resuscitation (CPR) attempts restoring spontaneous circulation [1, 2]. Of the survivals, an estimated 10–20% will ever regain meaningful neurologic recovery [1, 2]. For years, neurologists have used the landmark paper by Levy et al. to guide prognostication [3]. Included in this prognostication algorithm is myoclonus status epilepticus, which is typically regarded as predictive of a poor outcome, especially within the first 24 hours after cardiac arrest [3–5].

Myoclonus status epilepticus is a clinical diagnosis and is defined as spontaneous or sound sensitive, repetitive, irregular brief jerks of the face and limb for most of the first day after cardiac resuscitation [4]. It may be observed in up to 37% of patients following cardiac resuscitation [4]. With the increasing use of continuous electroencephalography (CEEG), underlying seizure patterns are being recognized [5]. Additionally, background CEEG patterns are being recognized as having prognostic value [6–8]. For example, in patients with myoclonus, the finding of burst suppression background with high amplitude polyspikes compared to continuous slowing with narrow, vertex spike-wave discharges may have worse prognosis [8]. However, the underlying cerebral activity can be difficult to interpret given the myogenic artifact that occurs with myoclonus obscuring the underlying CEEG background [9]. Additionally, recording uninterpretable CEEG can be costly to the healthcare system [10]. The use of a short-acting neuromuscular blocker in these patients can be useful for identifying background cerebral activity and may ultimately limit the duration of recording [11].

The purpose of this study was to evaluate change in quality of CEEG recording following neuromuscular blockade in patients admitted with cardiac arrest.

## 2. Methods

### 2.1. Patients

We retrospectively reviewed consecutive charts of postcardiac arrest patients over a 24-month period admitted to an academic medical center. Patients were identified for this study through the EEG database and neurocritical care consultation list. The patients were originally admitted in coma from anoxic brain injury. Patients were included in this study if they developed postanoxic myoclonus and were subsequently monitored on CEEG and received cisatracurium during their CEEG monitoring. All patients were normothermic during CEEG recording. This study was exempted from approval by the institutional review board because all subjects were deceased and the study analyzes data obtained as part of routine clinical practice.

### 2.2. Data Acquisition

Deidentified data were abstracted from the medical records from clinical notes, medication logs, imaging and diagnostic studies, and laboratory.

CEEG was recorded using 21 electrodes placed according to the International 10–20 System by certified EEG technologists and interpreted by board-certified electroencephalographers. Unless noted otherwise, CEEG was recorded in 15-second epochs using bitemporal montage at sensitivity of 5–7 uV/mm. Filters were set at 1 Hz (low frequency) and 70 Hz (high frequency). The 60 Hz notch was used. CEEG was noted to be partially or completely obscured by myogenic artifact. If partially obscured, the CEEG was reviewed for the following patterns: continuous or lateralized slow activity, generalized periodic discharge, lateralized periodic discharges (historically termed periodic lateralized epileptiform discharges (PLEDs)), burst suppression, and/or EEG seizures and/or status epilepticus. CEEG seizures were defined as evolving rhythms in frequency, distribution, and/or morphology at 3 Hz or greater for more than 10-second duration. Nonconvulsive status epilepticus was defined as continuous ictal pattern lasting > 30 minutes or ictal pattern present in more than 50% of 1 hour of CEEG.

### 2.3. Cisatracurium Administration Protocol

Prior to administration of cisatracurium, the respiratory rate and tidal volume were adjusted to maintain the minute ventilation. Sedation, if needed, was provided with fentanyl infusion. Once minute ventilation stabilized, cisatracurium was administered at a dose of 0.1–2 mg/kg while monitoring the CEEG. Repeat dosing was provided if myogenic artifact was still noted on CEEG.

### 2.4. Data Interpretation

Two reviewers (a neurologist and an epileptologist) blindly evaluated the CEEG tracings. CEEG was reviewed for initial frequencies prior to cisatracurium and frequencies after cisatracurium administration. Interrater agreement was then calculated. For comparison within one CEEG, the high frequency filter was adjusted to 70, 50, 30, 15, and 5 Hz before and after cisatracurium administration.

## 3. Results

### 3.1. Patient Characteristics

Data was collected on twelve patients with an average age of 53.25 years (range: 28–78 years). The majority were males (58.3%) with a history of hypertension (50%) and/or coronary artery disease (58.3%). The majority of arrests were pulseless electrical arrest (PEA)/asystole (83.3%) while ventricular fibrillation (VFib)/ventricular tachycardia (VTach) arrests accounted for 16.7%. All twelve patients expired in the hospital from withdrawal of life sustaining treatment. Patient characteristics are shown in Table 1.

### 3.2. EEG Characteristics

**Figure 1:**
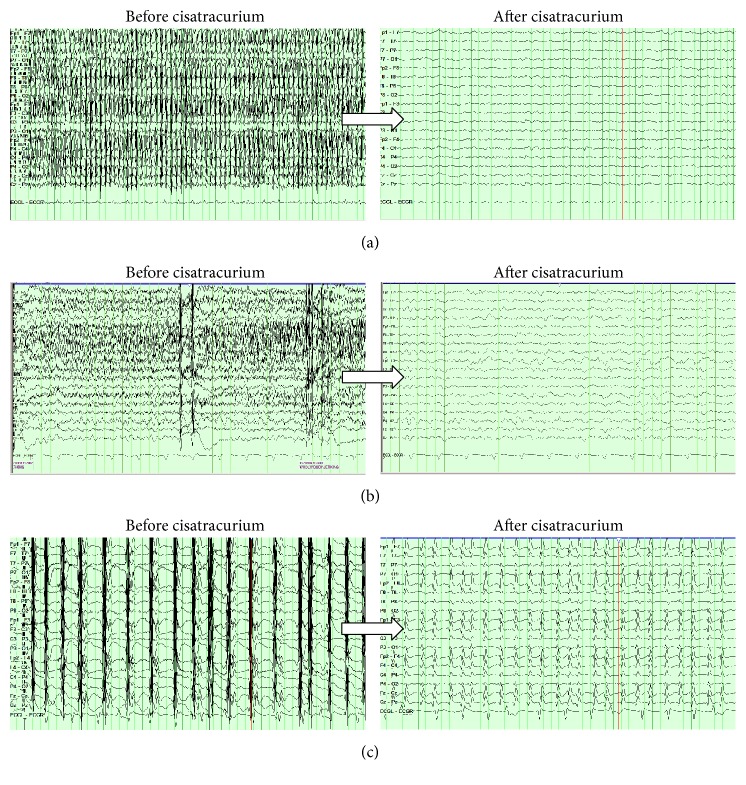
Effect of cisatracurium on continuous electroencephalography (CEEG). (a) The CEEG is completely obscured by myogenic artifact. After neuromuscular blockade, generalized continuous slowing is seen. (b) Myogenic artifact obscured the CEEG. After neuromuscular blockade, generalized slowing along with lateralized right slowing is seen. (c) Rhythmic myogenic artifact partially obscured the CEEG. After neuromuscular blockade, 3 Hz generalized periodic discharges were seen consistent with nonconvulsive status epilepticus.

Prior to neuromuscular blockade, the CEEG was interpreted as continuous slowing or burst suppression (*n* = 11; Table 2). The CEEG in one patient was completely obscured from myogenic artifact (Figure 1(a)). The interrater agreement of reduction in myogenic artifact allowing for better visualization of underlying cerebral activity was “perfect” (i.e., kapp = 1.00) between these CEEG recordings. “Perfect” (i.e., kappa = 1.00) interrater agreement was observed with precisatracurium CEEG interpretation.

After administration of cisatracurium, the interpretation of the CEEG was adjusted in nine patients. In five patients, the underlying EEG patterns were then recognized as either lateralized slowing, burst suppression, or generalized periodic discharges. The one patient with obscured CEEG from myogenic artifact was noted to have generalized, continuous slowing. Postcisatracurium interrater agreement was “perfect” (i.e., kappa = 1.00) for identifying lateralized slowing, burst suppression, and generalized periodic discharges. However, interrater agreement was only “moderate” for classifying seizure (i.e., kappa = 0.412) and generalized continuous slowing (i.e., kappa = 0.471).  Figure 1(b) is a representative CEEG showing the continuous slowing with lateralized right slowing. In three patients, underlying CEEG seizure activity was noted. Representative CEEG is shown in Figure 1(c). The CEEG patterns are shown in Table 2.

**Figure 2:**
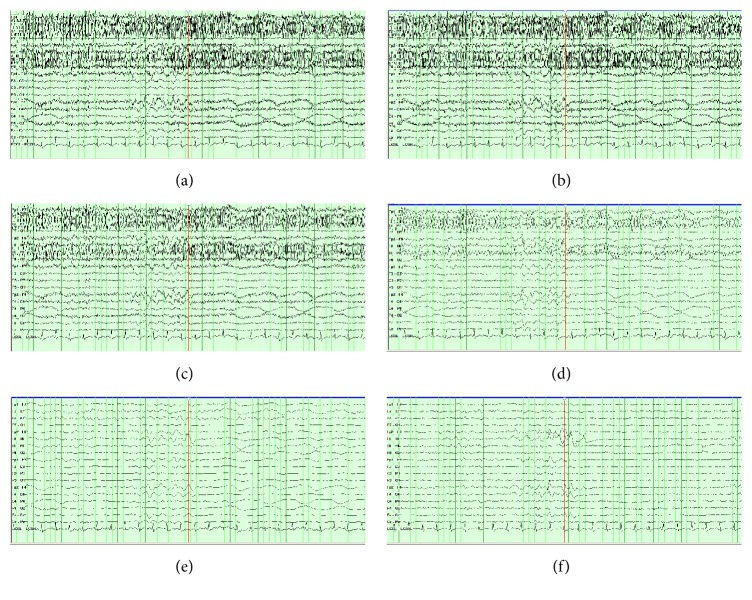
Effect of filtering continuous electroencephalography (CEEG) compared to cisatracurium. CEEG is partially obscured by myogenic artifact filtered at (a) 70 Hz, (b) 50 Hz, (c) 30 Hz, (d) 15 Hz, and (e) 5 Hz compared to neuromuscular blockade (f). This patient had asymmetric burst suppression (decreased left hemisphere).

For comparison, adjusting the high frequency filters of the CEEG in a patient with myogenic artifact shows that the faster myogenic artifact can be removed (Figures 2(a)–2(e)) but at the expense of losing faster frequencies (Figure 2(f)). This patient had asymmetric burst suppression (decreased left hemisphere) that was not well appreciated with filter adjustment.

## 4. Discussion

Determining cortical origin of myoclonus and background cerebral activity with CEEG can be difficult secondary to muscle artifact. We have shown in this study that the use of neuromuscular blockade in patients with postanoxic myoclonus is useful for identifying the background cerebral activity.

Myoclonic status epilepticus has historically been independently associated with poor outcome in coma patients after cardiac arrest [3, 4]. Recently, reports have shown that patients with postanoxic myoclonus can have good outcomes [12]. In a study by Bouwes et al., 12% of the patients with posthypoxic myoclonus had good neurological outcomes defined as Glasgow Outcome Scale (GOS) of 4 or 5 [12]. EEG may be useful to determine who may have favorable outcome.

Background activity on the CEEG has also been shown to have prognostic significance. Rossetti et al. studied 34 consecutive comatose patients treated with hypothermia after cardiac arrest [13]. Nonreactive background on CEEG was seen in 12/15 (75%) of nonsurvivors versus 0/19 (0%) in survivors [13]. Similarly, discontinuous “burst suppression” activity and EEG seizures with absent backgrounds were seen in in 11/15 (73%) and 7/15 (47%), respectively, in nonsurvivors compared to 0/19 in survivors [13]. No improvement in background reactivity or seizures/epileptiform discharges were seen once rewarmed [13]. All survivors had background CEEG reactivity, and majority (14/19, 74%) had a favorable outcome of cerebral performance score (CPC) 1 or 2 [14]. Routine EEGs of rewarmed, comatose patients in the Targeted Temperature Management trial showed various patterns: highly malignant (suppression, suppression with periodic discharges, and burst suppression), malignant (periodic or rhythmic patterns, pathological or nonreactive background), and benign (absence of malignant features) [15]. 37% of patients had a highly malignant EEG pattern, and all had a poor outcome (CPC of 3–5) [15]. Malignant EEG patterns had low specificity to predict poor prognosis (48%), but specificity increased if 2 or more malignant EEG patterns were present [15]. A benign EEG pattern was found in 1% of patients with a poor outcome [15]. Similarly, Elmer et al. found that postanoxic patients with myoclonus who have burst suppression background (i.e., pattern 1) had worse prognosis compared to those with a continuous background (i.e., pattern 2) [8]. Importantly, pattern 1 was found in 74% of the patients [8]. Of the patients who had pattern 1, no one survived, and only 50% of those who had pattern 2 survived [8]. Collectively, these studies highlight the importance of accurate interpretation of background cerebral reactivity on CEEG. Despite these results, none of the patients in our series survived to hospital discharge. This finding highlights the challenges of neurological prognostication after cardiac arrest and the self-fulfilling prophecy particularly since no index predicts poor neurological outcome with absolute certainty [16].

Myogenic artifact can obscure the CEEG, making it challenging to interpret the CEEG completely and, thus, accurately. Adjusting filters has been one method of reducing the artifact. However, this practice can alter the underlying EEG activity as we have shown. Algorithms have been created to remove the myogenic artifact [17]. It is still uncertain which algorithm performs optimally in a controlled environment [17]. The ICU is an environment with multiple sources of artifact which can make algorithms less faithful [17–19]. Accurately interpreting the background CEEG activity is important [8, 13–15]. Neuromuscular blockade can be used to reduce myogenic artifact allowing for clearer visualization of the underlying cerebral activity as we have demonstrated.

Whether aggressive treatment of myoclonus or CEEG seizures changes long-term outcomes is unclear. Seizures are common postcardiac arrest. In a review of our CEEG database, electrographic seizures occur in 26.7% of patients monitored with CEEG after anoxic injury (unpublished data, Christopher R. Newey). In the study by Bouwes et al., somatosensory evoked potentials (SSEP) and EEG were used to determine cortical or subcortical nature of the myoclonus [12]. Patient outcome was not correlated to origin of the myoclonus and was considered good outcome in 12% [12]. In contrast, a small study from Cincinnati found seizures in 33% of its cardiac arrest patients (11 of 33) with 9 of these patients expiring before discharge [20]. None survived by 30 days [20]. Similarly, all patients with seizures in a cohort from Mayo Clinic also had poor outcome [21]. Likewise, 94% of patients with epileptiform activity from a cohort from the University of Pennsylvania had poor neurologic outcome or death at discharge [22].

Recognizing that patients with myoclonus can have good neurological outcomes and knowing that seizures are commonly identified on CEEG, aggressive treatment of the seizure seems reasonable and may be a source for therapeutic opportunity to improve outcome [23]. The use of neuromuscular blocker on CEEG obscured by myogenic artifact can allow for recognition of cortical or subcortical origin of myoclonus and/or recognition of underlying seizure activity on CEEG.

Many centers have limited resources (personnel and equipment) and may have limitations with CEEG monitoring of cardiac arrest patients. In a study of cardiac arrest patients who underwent therapeutic hypothermia before and after CEEG monitoring protocol, 91 did not have CEEG by protocol and 62 patients had CEEG by protocol [10]. In those 91 patients before CEEG protocol, 19 patients had routine EEGs and 4 had CEEG at discretion of the attending physician [10]. The mean estimated CEEG charges for the pre-CEEG protocol cohort was $1571.59 per patient compared to $4214.93 per patient after CEEG protocol monitoring during therapeutic hypothermia [10]. Despite the addition of CEEG monitoring to the therapeutic hypothermia protocol, there was no difference in mortality [10]. Additionally, Alvarez et al. compared two 20-minute EEGs randomly extracted from a CEEG recording during therapeutic hypothermia and during normothermia [24]. Thirty-four recordings were studied, and they found agreement of 97.1% for background discontinuity and reactivity and 94.1% for epileptiform activity in therapeutic hypothermia [24]. In normothermia, there were no discrepancies [24]. Our study complements these studies by highlighting the ability to obtain background CEEG patterns with the use of a neuromuscular blocker which can limit the time needed to monitor postcardiac arrest patients. The ability to review background CEEG pattern early in the course of recording in cases of myoclonic artifact is cost-effective and has practical implications particularly if CEEG resources are limited.

Cisatracurium was chosen for neuromuscular blockade in our patient population. It has a unique mechanism of action for degradation via Hoffman elimination [25]. It is metabolized to laudanosine and ultimately excreted in the urine [26]. Since it does not rely on liver function for metabolism, it is ideally suited for use in postcardiac arrest patients who may have liver dysfunction [27]. The recommended bolus is 0.1–0.2 mg/kg with an onset of 90–120 seconds and duration of action of 45–75 minutes [27]. Prior to administration of neuromuscular blockade, the ventilator was adjusted to volume control ventilation followed by adjustment in respiratory rate and tidal volume to maintain minute ventilation. Sedation included fentanyl infusion. Benzodiazepines and propofol were held secondary to their known effects on CEEG and confounding neurological prognosis [5, 28]. Additionally, the hypotension that may occur with these agents, particularly in the critically ill, influenced the decision to use fentanyl alone [29].

This study is inherently limited by being a retrospective review of patients. Additionally, self-fulfilling prophecy cannot be excluded from studies such as this. It is not known if patients with seizures would have good neurological outcomes if treated aggressively. The clinical recognition of status myoclonus has been suggested to be predictive of poor outcome [4]. It is currently part of the AAN practice parameter guidelines as level B evidence supporting poor prognosis [4]. As such, all patients in this study had withdrawal of life sustaining treatment. Also, this study was not designed to review the cost analysis from decreased duration of CEEG after the use of cisatracurium. These should be studied further in a large, randomized controlled trial. We found variability in CEEG interpretation, especially with classifying seizures or continuous slowing. The interrater agreement for each of these was moderate. CEEG terminology has been evolving [30]. Interrater agreement in interpreting CEEG, especially with periodic discharges, has been a recognized problem [31, 32]. Board certification in Clinical Neurophysiology as well as the use of quantitative EEG has been associated with improved interrater agreement [33, 34]. As we learn more about the prognostic value of underlying background on CEEG, it is important that the interrater agreement for CEEG interpretation improves.

In conclusion, the use of neuromuscular blockade in patients with postanoxic myoclonus who are monitored on CEEG can be useful in identifying the background cortical activity. Accurately identifying background activity is known to be important in prognostication. Future studies should evaluate the cost analysis of continuous EEG recording with myogenic artifact with and without neuromuscular blockade in patients with postanoxic myoclonus.

## Figures and Tables

**Table 1:** Patient characteristics.

Total patients (*n*)	12	
Age (average, range; yrs)	53.25	28, 78
Male (*n*, %)	7	58.3
Past medical history (*n*, %)		
HTN	6	50.0
DMII	1	8.3
HLD	1	8.3
CKD	1	8.3
CAD	7	58.3
Cancer	4	33.3
Sepsis	2	16.7

Type of arrest (*n*, %)		
PEA/asystole	10	83.3
VFib/VTach	2	16.7

CAD, coronary artery disease; CKD, chronic kidney disease; DMII, diabetes mellitus type II; HLD, hyperlipidemia; HTN, hypertension; *n*, number; PEA, pulseless electrical activity; VFib, ventricular fibrillation; VTach, ventricular tachycardia; yrs, years.

**Table 2:** CEEG characteristics before and after neuromuscular blockade.

Before neuromuscular blockade	*N*	%
Continuous slowing +/− myogenic artifact	6	50.0
Burst suppression +/− myogenic artifact	5	41.7
Myogenic artifact obscuring EEG	1	8.3

After neuromuscular blockade	*N*	%

Change in CEEG interpretation	9	75.0
Patterns		
EEG seizure	3	33.3
Lateralized slowing	2	16.7
Burst suppression	2	16.7
Generalized periodic discharges	1	8.3
Continuous slowing (obscured EEG)	1	8.3

EEG, electroencephalography; *N*, number.
